# Supplementary material for: Deprotection of centromeric cohesin at meiosis II requires APC/C activity but not kinetochore tension
Source: EMBO J. 2021 Mar 1;40(7):e106812. doi: 10.15252/embj.2020106812 (PMC8013787; doi:10.15252/embj.2020106812)
Supplement: Supplementary file 1 — Appendix [file EMBJ-40-e106812-s002.pdf]

APPENDIX

Deprotection of Centromere Cohesin at Meiosis II Requires APC/C Activity but not Kinetochore Tension

Valentina Mengoli, Katarzyna Jonak, Oleksii Lyzak, Mahdi Lamb, Lisa M. Lister, Chris Lodge, Julie Rojas, Ievgeniia Zagoriy, Mary Herbert, and Wolfgang Zachariae

TABLE OF CONTENTS

Appendix Figure S1. The *SPO12* deletion delays APC/C-dependent proteolysis at meiosis II

Appendix Figure S2. *mam1Δ* mutants prolong metaphase I in a Mad2-dependent manner

Appendix Figure S3. Effect of the *SPO12* deletion on APC/C-dependent proteolysis in the *mad2Δ* mutant

Appendix Figure S4. Effects of deleting *SPO12* or *SPO12* and *MAM1* in *mad1Δ mad2Δ mad3Δ* triple mutants

Appendix Figure S5. Deletion of *SPO11* does not prolong metaphase I in the *mad2Δ* mutant

Appendix Table S1. *Saccharomyces cerevisiae* SK1 Strains Used in this Study

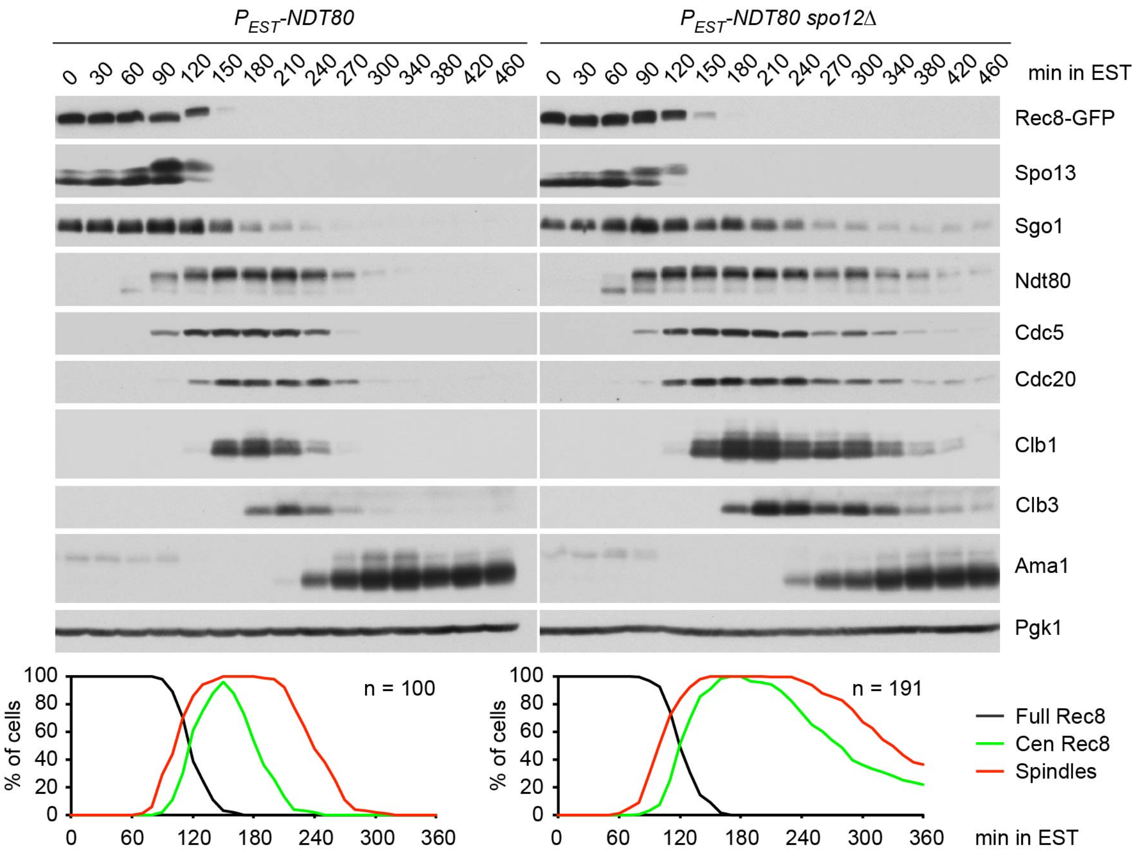

Appendix Figure S1. The *SPO12* deletion delays APC/C-dependent proteolysis at meiosis II

Control and *spo12Δ* strains synchronized with an estradiol-inducible *NDT80* gene (P_{EST}-*NDT80*) were analysed by immunoblot detection of proteins in whole cell extracts (panels). In parallel, Rec8-GFP and RFP-tubulin were followed by live-imaging (graphs). Deletion of *SPO12* delays degradation of APC/C substrates, removal of centromeric Rec8, and spindle disassembly at meiosis II by ~2 hr. Data are representative of 2 independent experiments.

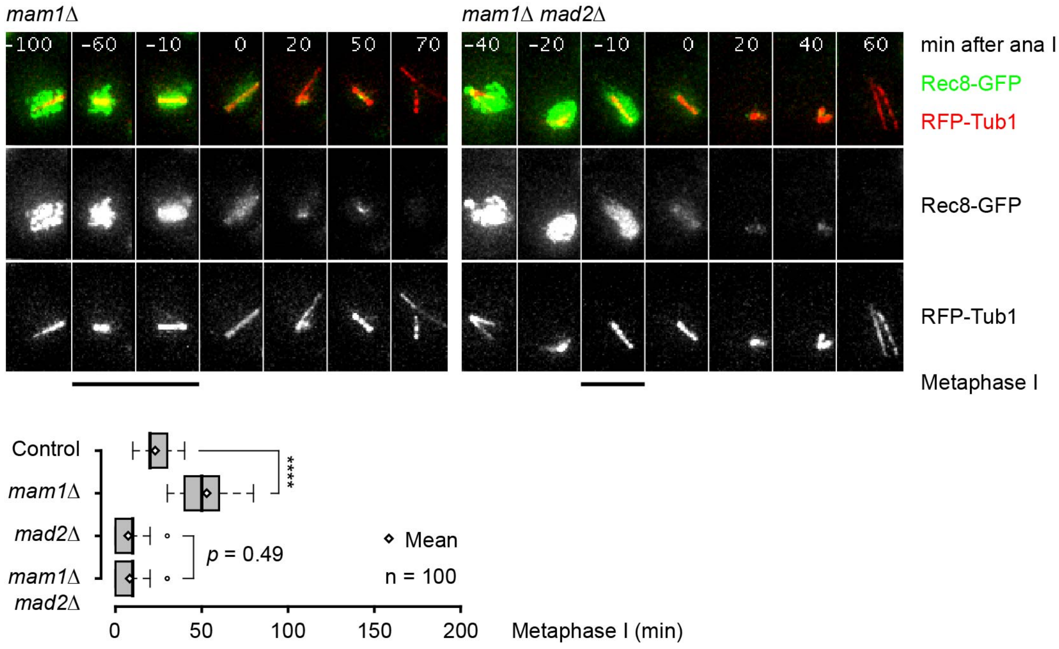

Appendix Figure S2. *mam1Δ* mutants prolong metaphase I in a Mad2-dependent manner

Imaging of Rec8-GFP and RFP-tubulin to determine the duration of metaphase I in control and *mam1Δ* cells containing or lacking Mad2. Top, representative time-lapse series. Bottom, duration of metaphase I (spindle formation to Rec8 cleavage at meiosis I) in the indicated strains. **** $p < 0.0001$, Mann-Whitney test. Data are representative of 2 independent experiments.

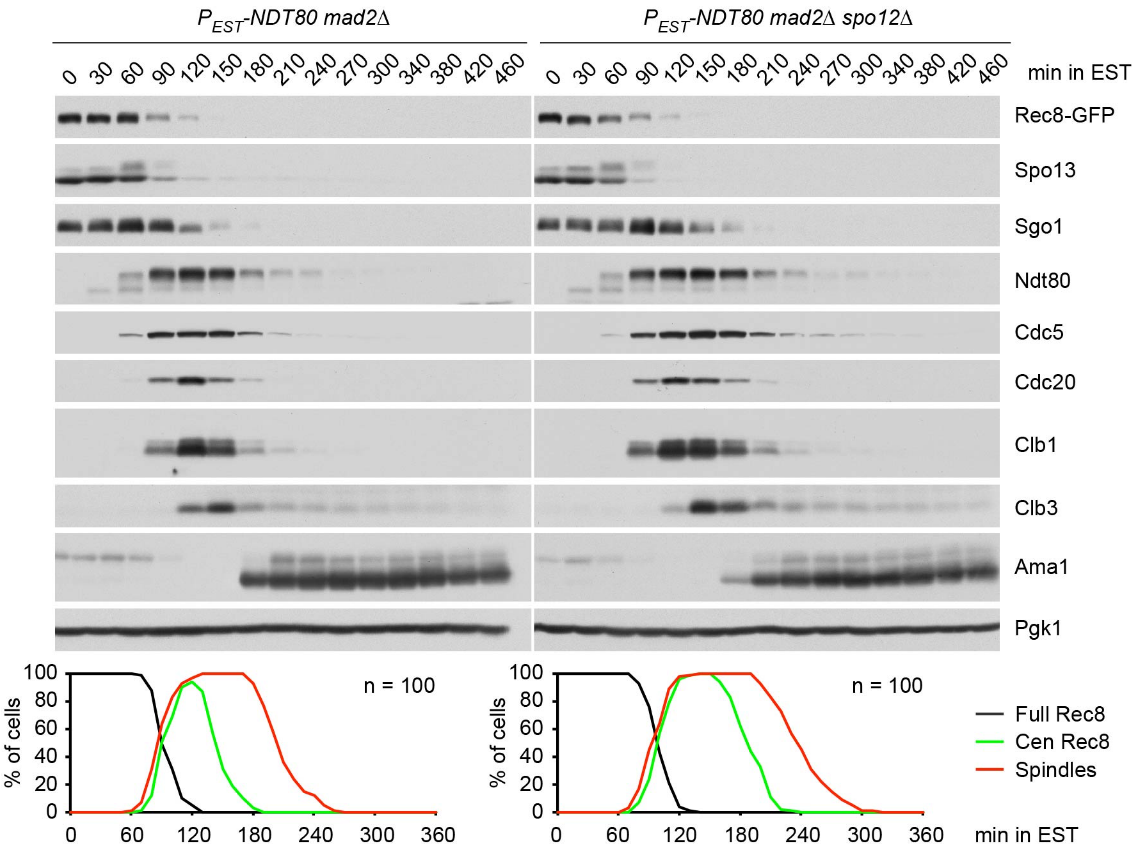

Appendix Figure S3. Effect of the *SPO12* deletion on APC/C-dependent proteolysis in the *mad2Δ* mutant

mad2Δ and *mad2Δ spo12Δ* strains synchronized with an estradiol-inducible *NDT80* gene (P_{EST}-*NDT80*) were analysed by immunoblot detection of proteins in whole cell extracts (panels). In parallel, Rec8-GFP and RFP-tubulin were followed by live-imaging (graphs). Deletion of *SPO12* in *mad2Δ* cells delays degradation of APC/C substrates, removal of centromeric Rec8, and spindle disassembly at meiosis II by only ~0.5 hr. Data are representative of 2 independent experiments.

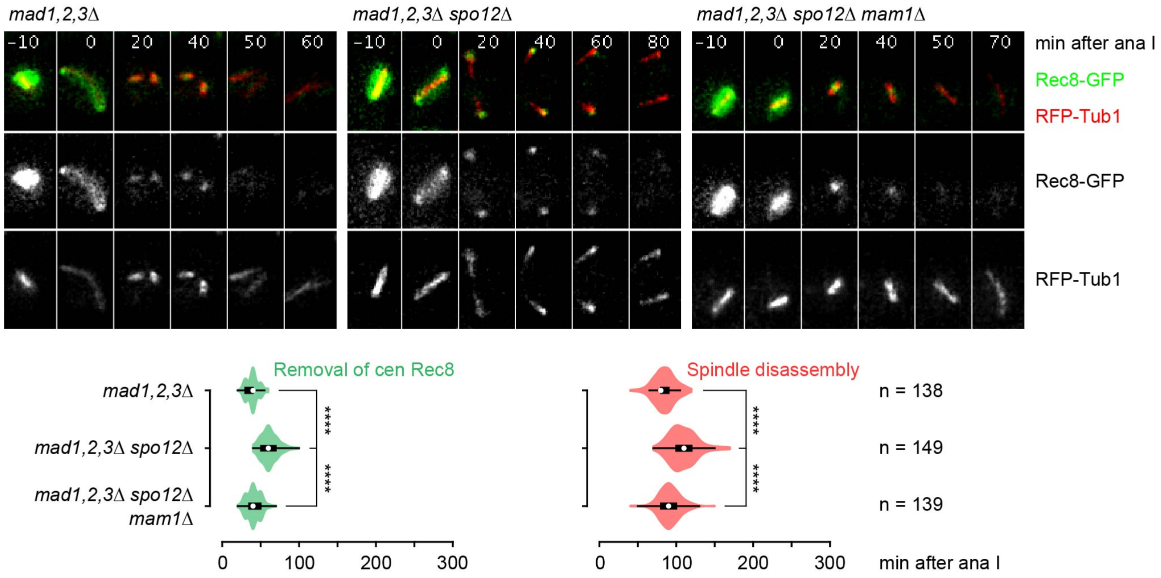

Appendix Figure S4. Effects of deleting *SPO12* or *SPO12* and *MAM1* in *mad1Δ mad2Δ mad3Δ* triple mutants

Top, time-lapse series from the imaging of Rec8-GFP and RFP-tubulin. Bottom, times from cohesin cleavage at anaphase I to the removal of centromeric Rec8 and to spindle disassembly. **** $p < 0.0001$, Mann-Whitney test. Data are representative of 2 independent experiments.

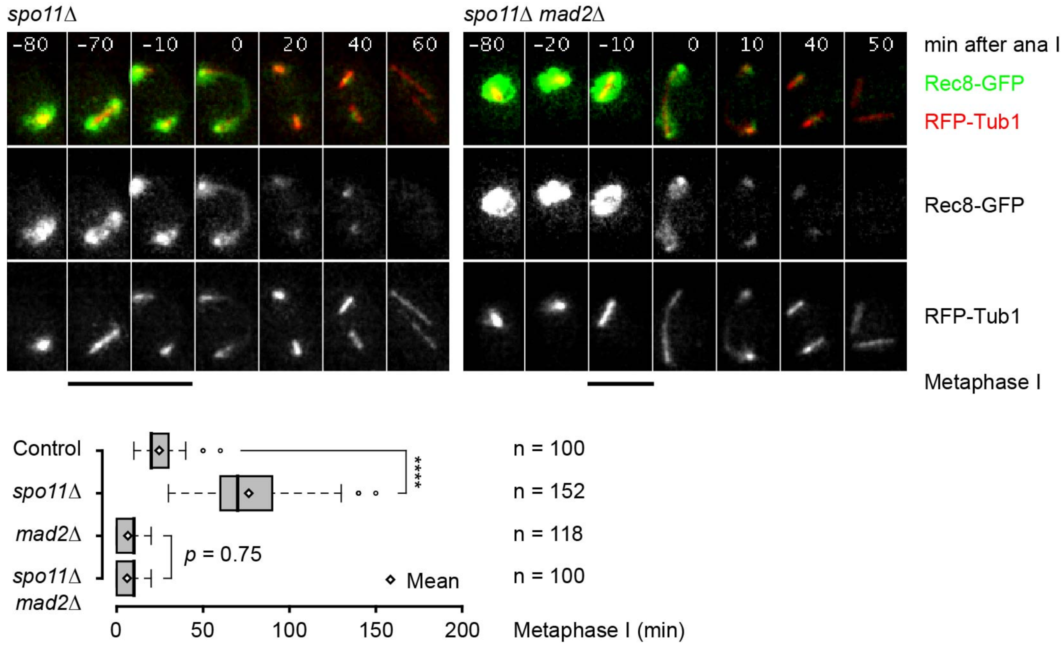

Appendix Figure S5. Deletion of *SPO11* does not prolong metaphase I in the *mad2Δ* mutant

Imaging of Rec8-GFP and RFP-tubulin to determine the duration of metaphase I in control and *spo11Δ* cells containing or lacking Mad2. Top, representative time-lapse series. Bottom, duration of metaphase I (spindle formation to Rec8 cleavage at meiosis I) in the indicated strains. **** $p < 0.0001$, Mann-Whitney test.

Appendix Table S1. ***Saccharomyces cerevisiae*** **SK1 Strains Used in this Study**

Figure	Strain[1]	Genotype[2]
1B	Z32945	*rec8Δ::KanMX4::REC8-mNeonGreen::LEU2 ura3::*P_{HIS3}*-mCherry-TUB1::URA3*
1B	Z32944	*rec8Δ::KanMX4::REC8-mNeonGreen::LEU2 ura3::*P_{HIS3}*-mCherry-TUB1::URA3 spo12Δ::NatMX4*
1C	Z33612	*rec8Δ::KanMX4::REC8-mNeonGreen::LEU2 CEN5/CEN5::*$tetO_{224}$*::HIS3 leu2/leu2::*P_{URA3}*-tetR-tdTomato::LEU2*
1C	Z33613	*rec8Δ::KanMX4::REC8-mNeonGreen::LEU2 CEN5/CEN5::*$tetO_{224}$*::HIS3 leu2/leu2::*P_{URA3}*-tetR-tdTomato::LEU2 spo12Δ::NatMX4*
1D	Z33261	*MAD2-mNeonGreen::KlTRP1 CNM67-tdTomato::NatMX4*
1D	Z33262	*MAD2-mNeonGreen::KlTRP1 CNM67-tdTomato::NatMX4 spo12Δ::NatMX4*
2B	Z35330	*rec8Δ::KanMX4::REC8-mNeonGreen::LEU2 ura3::*P_{HIS3}*-mCherry-TUB1::URA3 mam1Δ::HIS3*
2B	Z35329	*rec8Δ::KanMX4::REC8-mNeonGreen::LEU2 ura3::*P_{HIS3}*-mCherry-TUB1::URA3 mam1Δ::HIS3 spo12Δ::NatMX4*
2C	Z33614	*rec8Δ::KanMX4::REC8-mNeonGreen::LEU2 CEN5/CEN5::*$tetO_{224}$*::HIS3 leu2/leu2::*P_{URA3}*-tetR-tdTomato::LEU2 mam1Δ::HIS3*
2C	Z33615	*rec8Δ::KanMX4::REC8-mNeonGreen::LEU2 CEN5/CEN5::*$tetO_{224}$*::HIS3 leu2/leu2::*P_{URA3}*-tetR-tdTomato::LEU2 mam1Δ::HIS3 spo12Δ::NatMX4*
2D	Z33264	*MAD2-mNeonGreen::KlTRP1 CNM67-tdTomato::NatMX4 mam1Δ::HIS3*
2D	Z33262	*MAD2-mNeonGreen::KlTRP1 CNM67-tdTomato::NatMX4 mam1Δ::HIS3 spo12Δ::NatMX4*
3A	Z33827	*rec8Δ::KanMX4::REC8-mNeonGreen::LEU2 CEN5/CEN5::*$tetO_{224}$*::HIS3 leu2/leu2::*P_{URA3}*-tetR-tdTomato::LEU2 mad2Δ::KlURA3*
3A	Z33828	*rec8Δ::KanMX4::REC8-mNeonGreen::LEU2 CEN5/CEN5::*$tetO_{224}$*::HIS3 leu2/leu2::*P_{URA3}*-tetR-tdTomato::LEU2 mad2Δ::KlURA3 spo12Δ::NatMX4*
3A	Z33948	*rec8Δ::KanMX4::REC8-mNeonGreen::LEU2 CEN5/CEN5::*$tetO_{224}$*::HIS3 leu2/leu2::*P_{URA3}*-tetR-tdTomato::LEU2 mad2Δ::KlURA3 spo12Δ::NatMX4 mam1Δ::HIS3*
3B	Z30453	*rec8Δ::KanMX4::REC8-mNeonGreen::LEU2 ura3::*P_{HIS3}*-mCherry-TUB1::URA3 mad2Δ::KlURA3*
3B	Z30454	*rec8Δ::KanMX4::REC8-mNeonGreen::LEU2 ura3::*P_{HIS3}*-mCherry-TUB1::URA3 mad2Δ::KlURA3 spo12Δ::NatMX4*
3B	Z30618	*rec8Δ::KanMX4::REC8-mNeonGreen::LEU2 ura3::*P_{HIS3}*-mCherry-TUB1::URA3 mad2Δ::KlURA3 spo12Δ::NatMX4 mam1Δ::HIS3*
3C	Z34176	*RTS1-eGFP::KanMX4 MTW1-mCherry::HphMX4 mad2Δ::KlURA3 spo12Δ::NatMX4*
3C	Z33507	*rec8Δ::KanMX4::REC8-mNeonGreen::LEU2 MTW1-mCherry::HphMX4 mad2Δ::KlURA3 spo12Δ::NatMX4*
3D	Z35525	*RTS1-mNeonGreen::KlTRP1 rec8Δ::KanMX4::REC8-mScarlet::LEU2 mad2Δ::KlURA3*
3D	Z35526	*RTS1-mNeonGreen::KlTRP1 rec8Δ::KanMX4::REC8-mScarlet::LEU2 mad2Δ::KlURA3 spo12Δ::NatMX4*
3D	Z35527	*RTS1-mNeonGreen::KlTRP1 rec8Δ::KanMX4::REC8-mScarlet::LEU2 mad2Δ::KlURA3 spo12Δ::NatMX4 mam1Δ::HIS3*
4A, B	Z27968	*cdc20::*P_{CLB2}*-CDC20::KanMX6 trp1::*P_{CUP1}*-CDC20::TRP1 ama1Δ::NatMX4 leu2::*P_{DMC1}*-cAMA1::LEU2 PDS1-myc18::HIS3MX6*

4A, B	Z31712	cdc20::P_{CLB2}-CDC20::KanMX6 trp1::P_{CUP1}-cdc20-3::TRP1 ama1Δ::NatMX4 leu2::P_{DMC1}-cAMA1::LEU2 PDS1-myc18::HIS3MX6
4C	Z37459	cdc20::P_{HSL1}-CDC20::HphMX4 trp1/trp1::P_{CUP1}-cdc20-3::TRP1 ama1Δ::NatMX4 leu2::P_{DMC1}-cAMA1::LEU2 RTS1-eGFP::BleMX4 MTW1-mCherry::HphMX4
4C	Z37470	cdc20::P_{HSL1}-CDC20::HphMX4 trp1/trp1::P_{CUP1}-cdc20-3::TRP1 ama1Δ::NatMX4 leu2::P_{DMC1}-cAMA1::LEU2 rec8Δ::KanMX4::REC8-mNeonGreen::LEU2 MTW1-mCherry::HphMX4
5A, B	Z36435	cdc20::P_{HSL1}-CDC20::HphMX4 trp1::P_{CUP1}-cdc20-3::TRP1 ama1Δ::NatMX4 leu2::P_{DMC1}-cAMA1::LEU2 REC8-ha3::URA3 PDS1-AID*::KanMX4
5A, B	Z36436	cdc20::P_{HSL1}-CDC20::HphMX4 trp1::P_{CUP1}-cdc20-3::TRP1 ama1Δ::NatMX4 leu2::P_{DMC1}-cAMA1::LEU2 REC8-ha3::URA3 PDS1-AID*::KanMX4 mps1-as
5A, B	Z35713	cdc20::P_{HSL1}-CDC20::HphMX4 trp1::P_{CUP1}-cdc20-3::TRP1 ama1Δ::NatMX4 leu2::P_{DMC1}-cAMA1::LEU2 REC8-ha3::URA3 PDS1-AID*::KanMX4 ura3::P_{CUP1}-OsTIR-myc3::URA3
5A, B	Z35714	cdc20::P_{HSL1}-CDC20::HphMX4 trp1::P_{CUP1}-cdc20-3::TRP1 ama1Δ::NatMX4 leu2::P_{DMC1}-cAMA1::LEU2 REC8-ha3::URA3 PDS1-AID*::KanMX4 mps1-as ura3::P_{CUP1}-OsTIR-myc3::URA3
6A, B	Z37076	cdc20::P_{HSL1}-CDC20::HphMX4 trp1::P_{CUP1}-cdc20-3::TRP1 ama1Δ::NatMX4 leu2::P_{DMC1}-cAMA1::LEU2 REC8-ha3::URA3 SGO1-AID*::KanMX4
6A, B	Z37077	cdc20::P_{HSL1}-CDC20::HphMX4 trp1::P_{CUP1}-cdc20-3::TRP1 ama1Δ::NatMX4 leu2::P_{DMC1}-cAMA1::LEU2 REC8-ha3::URA3 SGO1-AID*::KanMX4 ura3::P_{CUP1}-OsTIR-myc3::URA3
6C, D	Z37078	cdc20::P_{HSL1}-CDC20::HphMX4 trp1::P_{CUP1}-cdc20-3::TRP1 ama1Δ::NatMX4 leu2::P_{DMC1}-cAMA1::LEU2 REC8-ha3::URA3 SGO1-AID*::KanMX4 PDS1-AID*::KanMX4
6C, D	Z37079	cdc20::P_{HSL1}-CDC20::HphMX4 trp1::P_{CUP1}-cdc20-3::TRP1 ama1Δ::NatMX4 leu2::P_{DMC1}-cAMA1::LEU2 REC8-ha3::URA3 SGO1-AID*::KanMX4 PDS1-AID*::KanMX4 ura3::P_{CUP1}-OsTIR-myc3::URA3
EV1A	Z33310	SPC42/SPC42-eGFP::HIS3MX6 ura3::P_{HIS3}-mCherry-TUB1::URA3
EV1A	Z33309	SPC42/SPC42-eGFP::HIS3MX6 ura3::P_{HIS3}-mCherry-TUB1::URA3 spo12Δ::NatMX4
EV1B	Z33305	rec8Δ::KanMX4::REC8-mNeonGreen::LEU2 MTW1-mCherry::HphMX4
EV1B	Z33506	rec8Δ::KanMX4::REC8-mNeonGreen::LEU2 MTW1-mCherry::HphMX4 spo12Δ::NatMX4
EV1C	Z15736	RTS1-eGFP::KanMX4 MTW1-mCherry::HphMX4
EV1C	Z34178	RTS1-eGFP::KanMX4 MTW1-mCherry::HphMX4 spo12Δ::NatMX4
EV1D	Z34052	RTS1-mNeonGreen::KlTRP1 rec8Δ::KanMX4::REC8-mScarlet::LEU2
EV1D	Z34053	RTS1-mNeonGreen::KlTRP1 rec8Δ::KanMX4::REC8-mScarlet::LEU2 spo12Δ::NatMX4
EV2A	Z34890	REC8-ha3::URA3 CEN5/CEN5::tetO$_{224}$::HIS3 leu2/leu2::P_{URA3}-tetR-eGFP::LEU2 mad2Δ::KlURA3
EV2A	Z34891	REC8-ha3::URA3 CEN5/CEN5::tetO$_{224}$::HIS3 leu2/leu2::P_{URA3}-tetR-eGFP::LEU2 mad2Δ::KlURA3 spo12Δ::NatMX4
EV2B	Z37473	REC8-ha3::URA3 BMH1/BMH1:: tetO$_{224}$::URA3 leu2/leu2::P_{URA3}-tetR-eGFP::LEU2 mad2Δ::KlURA3
EV2B	Z37472	REC8-ha3::URA3 BMH1/BMH1:: tetO$_{224}$::URA3 leu2/leu2::P_{URA3}-tetR-eGFP::LEU2 mad2Δ::KlURA3 spo12Δ::NatMX4

EV2C	Z34907	ndt80Δ::NatMX4 leu2::P_{GAL1}-NDT80::LEU2 his3::P_{GPD}-$GAL4^{484}$-ER::HIS3 rec8Δ::KanMX4::REC8-mNeonGreen::LEU2 ura3::P_{HIS3}-mCherry-TUB1::URA3 mad2Δ::KlURA3
EV2C	Z34909	ndt80Δ::NatMX4 leu2::P_{GAL1}-NDT80::LEU2 his3::P_{GPD}-$GAL4^{484}$-ER::HIS3 rec8Δ::KanMX4::REC8-mNeonGreen::LEU2 ura3::P_{HIS3}-mCherry-TUB1::URA3 mad2Δ::KlURA3 trp1::P_{CUP1}-CDC20::TRP1
EV2C	Z34908	ndt80Δ::NatMX4 leu2::P_{GAL1}-NDT80::LEU2 his3::P_{GPD}-$GAL4^{484}$-ER::HIS3 rec8Δ::KanMX4::REC8-mNeonGreen::LEU2 ura3::P_{HIS3}-mCherry-TUB1::URA3 mad2Δ::KlURA3 spo12Δ::NatMX4
EV2C	Z34910	ndt80Δ::NatMX4 leu2::P_{GAL1}-NDT80::LEU2 his3::P_{GPD}-$GAL4^{484}$-ER::HIS3 rec8Δ::KanMX4::REC8-mNeonGreen::LEU2 ura3::P_{HIS3}-mCherry-TUB1::URA3 mad2Δ::KlURA3 spo12Δ::NatMX4 trp1::P_{CUP1}-CDC20::TRP1
EV3 A, B	Z35712	cdc20::P_{HSL1}-CDC20::HphMX4 trp1::P_{CUP1}-cdc20-3::TRP1 ama1Δ::NatMX4 leu2::P_{DMC1}-cAMA1::LEU2 REC8-ha3::URA3 PDS1-AID*::KanMX4
EV4	Z23511	cdc20::P_{CLB2}-CDC20::HphMX4 trp1::P_{CUP1}-CDC20::TRP1 his3::P_{GPD}-$GAL4^{484}$-ER::HIS3 ura3::P_{GAL1}-Ha3-REC8::URA3 PDS1-myc18::KlTRP1 gal4Δ::NatMX4
EV4	Z23496	cdc20::P_{CLB2}-CDC20::HphMX4 trp1::P_{CUP1}-CDC20::TRP1 his3::P_{GPD}-$GAL4^{484}$-ER::HIS3 ura3::P_{GAL1}-Ha3-rec8-24A::URA3 PDS1-myc18::KlTRP1 gal4Δ::NatMX4
S1	Z33877	ndt80Δ::NatMX4 leu2::P_{GAL1}-NDT80::LEU2 his3::P_{GPD}-$GAL4^{484}$-ER::HIS3 rec8Δ::KanMX4::REC8-mNeonGreen::LEU2 ura3::P_{HIS3}-mCherry-TUB1::URA3
S1	Z33878	ndt80Δ::NatMX4 leu2::P_{GAL1}-NDT80::LEU2 his3::P_{GPD}-$GAL4^{484}$-ER::HIS3 rec8Δ::KanMX4::REC8-mNeonGreen::LEU2 ura3::P_{HIS3}-mCherry-TUB1::URA3 spo12Δ::NatMX4
S2	Z36167	rec8Δ::KanMX4::REC8-mNeonGreen::LEU2 ura3::P_{HIS3}-mCherry-TUB1::URA3
S2	Z36169	rec8Δ::KanMX4::REC8-mNeonGreen::LEU2 ura3::P_{HIS3}-mCherry-TUB1::URA3 mam1Δ::HIS3
S2	Z36168	rec8Δ::KanMX4::REC8-mNeonGreen::LEU2 ura3::P_{HIS3}-mCherry-TUB1::URA3 mad2Δ::KlURA3
S2	Z36170	rec8Δ::KanMX4::REC8-mNeonGreen::LEU2 ura3::P_{HIS3}-mCherry-TUB1::URA3 mam1Δ::HIS3 mad2Δ::KlURA3
S3	Z34026	ndt80Δ::NatMX4 leu2::P_{GAL1}-NDT80::LEU2 his3::P_{GPD}-$GAL4^{484}$-ER::HIS3 rec8Δ::KanMX4::REC8-mNeonGreen::LEU2 ura3::P_{HIS3}-mCherry-TUB1::URA3 mad2Δ::KlURA3
S3	Z34025	ndt80Δ::NatMX4 leu2::P_{GAL1}-NDT80::LEU2 his3::P_{GPD}-$GAL4^{484}$-ER::HIS3 rec8Δ::KanMX4::REC8-mNeonGreen::LEU2 ura3::P_{HIS3}-mCherry-TUB1::URA3 mad2Δ::KlURA3 spo12Δ::NatMX4
S4	Z35132	rec8Δ::KanMX4::REC8-mNeonGreen::LEU2 ura3::P_{HIS3}-mCherry-TUB1::URA3 mad1Δ::KanMX4 mad2Δ::KlURA3 mad3Δ::KlTRP1
S4	Z35133	rec8Δ::KanMX4::REC8-mNeonGreen::LEU2 ura3::P_{HIS3}-mCherry-TUB1::URA3 mad1Δ::KanMX4 mad2Δ::KlURA3 mad3Δ::KlTRP1 spo12Δ::NatMX4
S4	Z35134	rec8Δ::KanMX4::REC8-mNeonGreen::LEU2 ura3::P_{HIS3}-mCherry-TUB1::URA3 mad1Δ::KanMX4 mad2Δ::KlURA3 mad3Δ::KlTRP1 spo12Δ::NatMX4 mam1Δ::HIS3
S5	Z32945	rec8Δ::KanMX4::REC8-mNeonGreen::LEU2 ura3::P_{HIS3}-mCherry-TUB1::URA3

S5	Z33724	*rec8Δ::KanMX4::REC8-mNeonGreen::LEU2 ura3::*P_{HIS3}*-mCherry-TUB1::URA3 spo11Δ::NatMX4*
S5	Z30453	*rec8Δ::KanMX4::REC8-mNeonGreen::LEU2 ura3::*P_{HIS3}*-mCherry-TUB1::URA3 mad2Δ::KlURA3*
S5	Z33723	*rec8Δ::KanMX4::REC8-mNeonGreen::LEU2 ura3::*P_{HIS3}*-mCherry-TUB1::URA3 spo11Δ::NatMX4 mad2Δ::KlURA3*

[1]Strains are listed for each figure from left to right and/or top to bottom. [2]All SK1 strains are diploid with the background *MATa/MATα ho::LYS2 lys2 ade2Δ::hisG trp1Δ::hisG leu2Δ::hisG his3Δ::hisG ura3*. Mutations are homozygous unless stated otherwise.
