# Supplementary material for: Deprotection of centromeric cohesin at meiosis II requires APC/C activity but not kinetochore tension
Source: EMBO J. 2021 Mar 1;40(7):e106812. doi: 10.15252/embj.2020106812 (PMC8013787; doi:10.15252/embj.2020106812)
Supplement: Supplementary file 2 — Expanded View Figures PDF [file EMBJ-40-e106812-s001.pdf]

Expanded View Figures

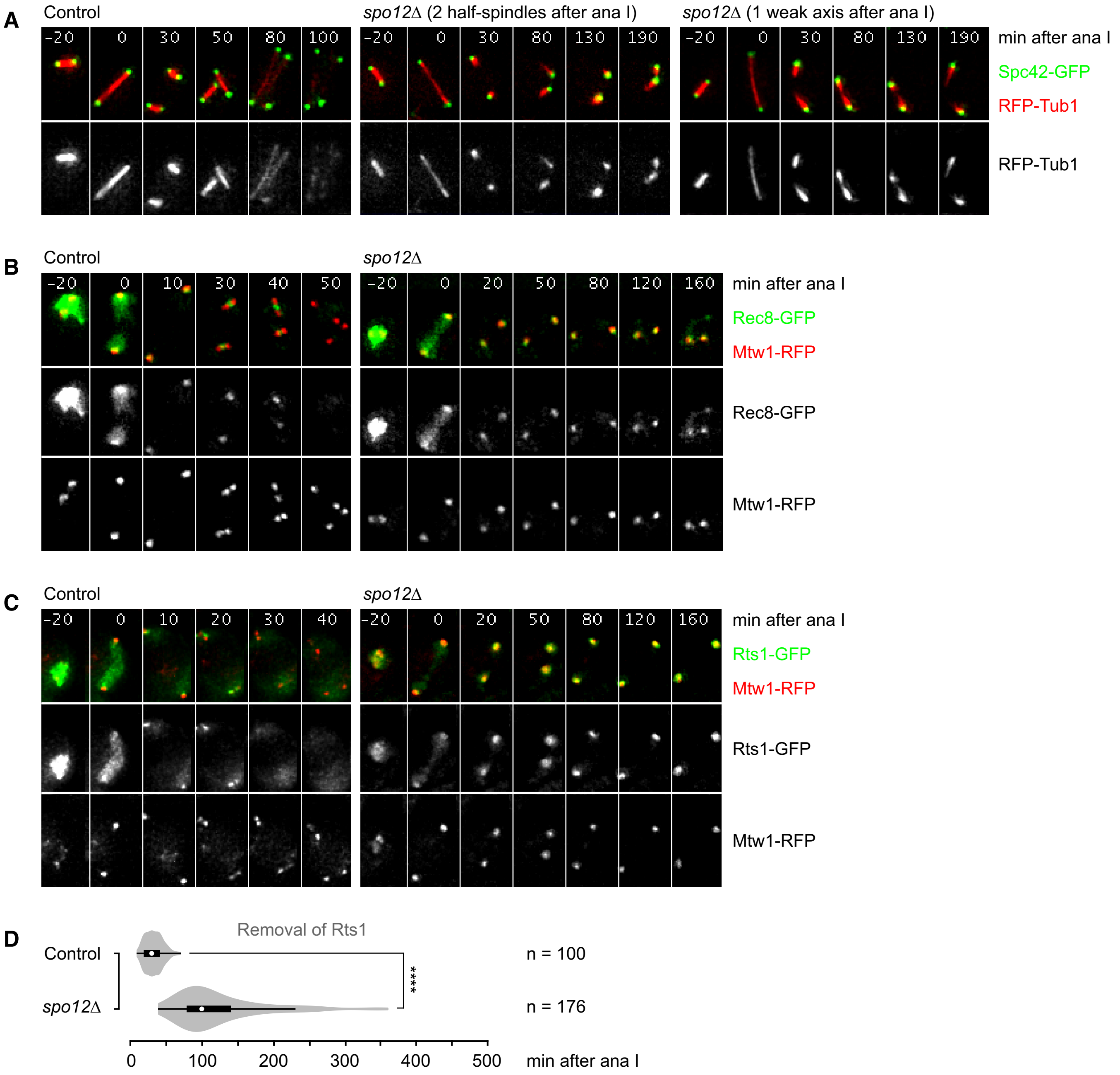

Figure EV1. Analysis of *spo12Δ* cells.

A Time-lapse series from the imaging of SPBs (Spc42-GFP) and RFP-tubulin in control (n = 100) and *spo12Δ* cells (n = 130). After anaphase I, *spo12Δ* cells (> 98%) fail to reduplicate SPBs and show two half-spindles (44%) or a spindle axis with a weak midzone (56%).

B Time-lapse series from the imaging of Rec8-GFP and kinetochores (Mtw1-RFP) in control and *spo12Δ* cells.

C Time-lapse series from the imaging of Rts1-GFP and kinetochores (Mtw1-RFP) in control and *spo12Δ* cells. Control cells were exposed 2× longer than *spo12Δ* cells.

D Imaging of Rts1-GFP and Rec8-RFP was used to measure the times from cohesin cleavage at anaphase I to the removal of Rts1 from kinetochores in control and *spo12Δ* cells. ****P < 0.0001, M–W test.

Source data are available online for this figure.

© 2021 The Authors

Figure EV2. Analysis of the *SPO12* deletion in cells lacking Mad2.

A, B Chromatin spreads from *mad2Δ* and *mad2Δ spo12Δ* cells were stained for SPBs (γ-tubulin), Rec8-ha3, and GFP dots marking sister sequences at the centromere (*CEN5*-GFP) or 394 kb from the centromere (*BMH1*-GFP) of chromosome 5. (A) Analysis of *CEN5*-GFP sister dots. (B) Analysis of *BMH1*-GFP sister dots. The histogram shows distances between sister dots in *mad2Δ spo12Δ* cells at anaphase II normalized to the diameter of a circle around the nucleus harboring the sister dots. Blue lines: mean values (*CEN5*-GFP, 0.08 ± 0.09; *BMH1*-GFP, 0.32 ± 0.18). Data were compared using the M–W test. We excluded cells that segregate *BMH1*-GFP dots to different nuclei as a result of recombination.

C Overexpression of *CDC20* at meiosis II in *mad2Δ* and *mad2Δ spo12Δ* cells. Strains were released from a prophase-arrest with an estradiol-inducible *NDT80* gene (P_{EST}-*NDT80*), and *CDC20* was overexpressed at late anaphase I using the *CUP1* promoter. Top, time-lapse series from the imaging of Rec8-GFP and RFP-tubulin. Bottom, times from cohesin cleavage at anaphase I to the removal of centromeric Rec8 and to spindle disassembly. ****$P < 0.0001$, M–W test. Data are representative of two independent experiments.

Source data are available online for this figure.

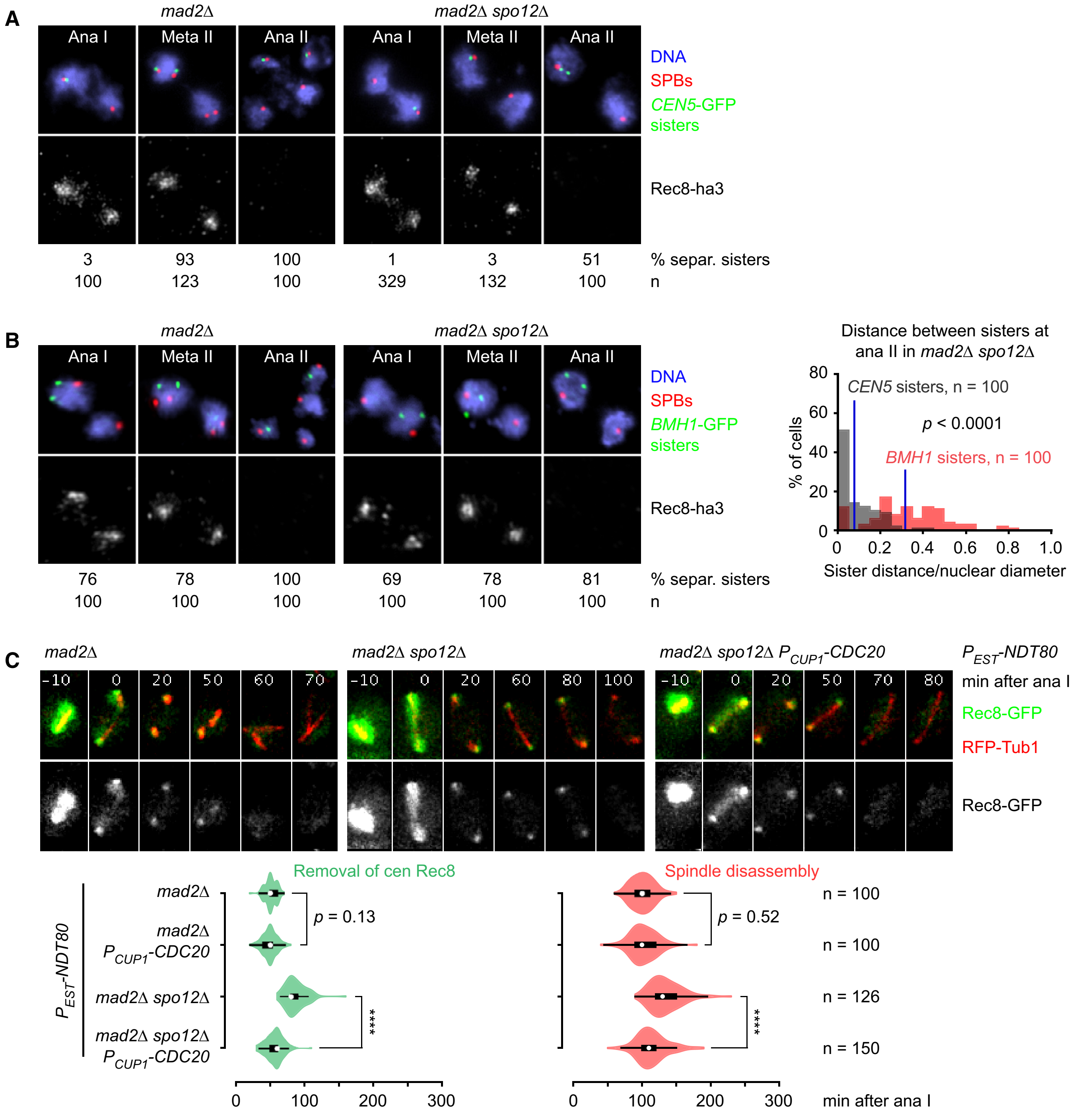

Figure EV2.

 © 2021 The Authors

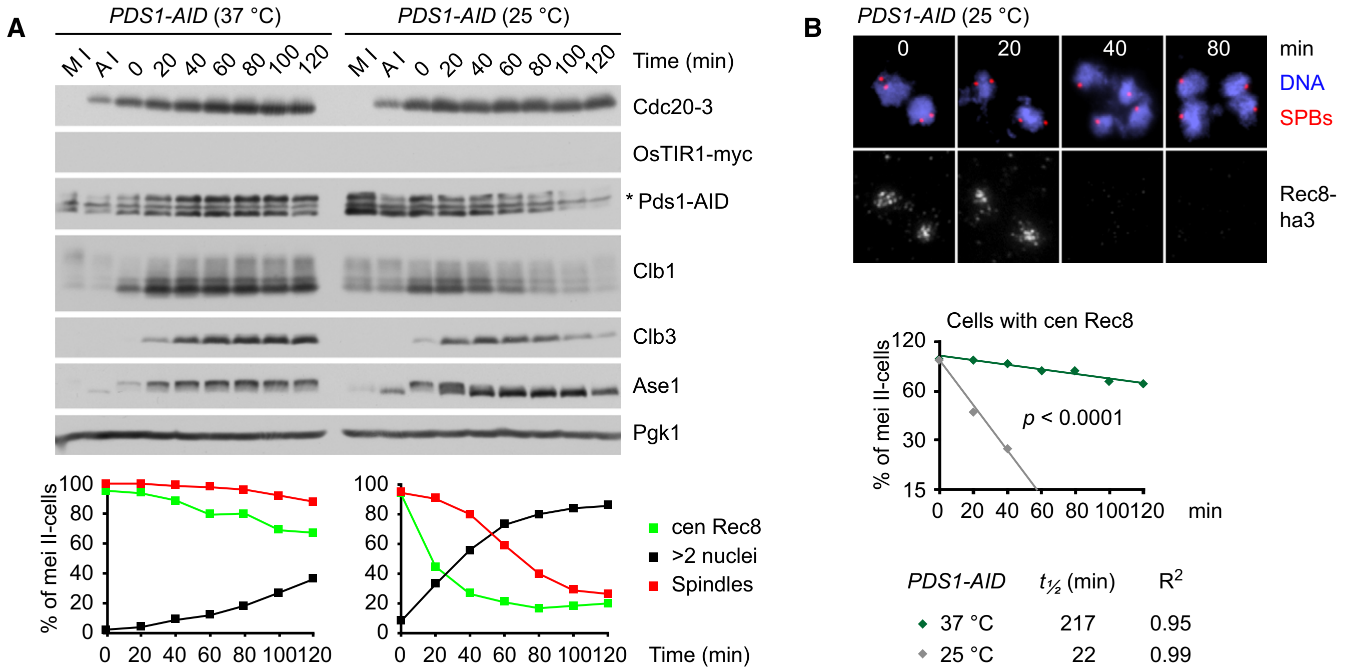

Figure EV3. Release of *cdc20^ts-mAR ama1* cells from the metaphase II arrest into anaphase II.

A, B *cdc20^ts-mAR ama1 PDS1-AID* cultures were released from metaphase I (M I) into anaphase (A I) at 25°C and then arrested at metaphase II by a shift to 37°C. At $t = 0$, cultures were treated with 1NM-PP1 and IAA and then either kept at 37°C or shifted back to 25°C. Drugs were added for consistency with Fig 5 but have no effect on this strain. (A) Top, protein plots. Bottom, percentages of meiosis II cells (four SPBs) with spindles, nuclear division (> 2 nuclei) and centromeric Rec8 (from chromatin spreads). (B) Top, chromatin spreads from meiosis II (four SPBs) stained for DNA, γ-tubulin/SPBs, and Rec8-ha3. Bottom, semi-log plot of the percentages of meiosis II cells with centromeric Rec8. Half-lives were calculated from exponential regression. Slopes were compared with ANCOVA.

Source data are available online for this figure.

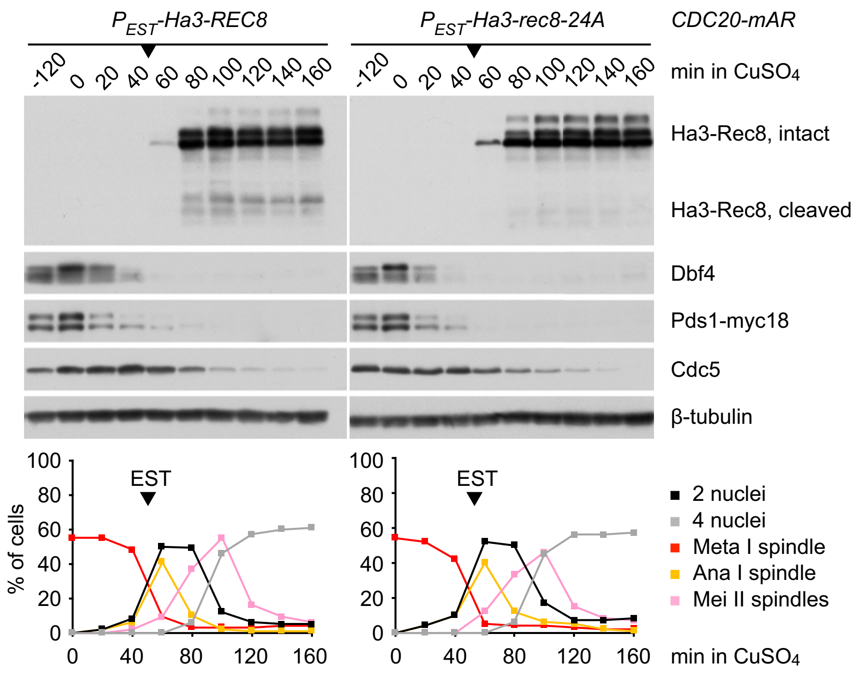

Figure EV4. Expression of non-cleavable Rec8 at anaphase I.

CDC20-mAR cells released from the metaphase I-arrest (+ $CuSO_4$, $t = 0$) were treated with estradiol ($t = 50$ min, arrows) to express wild-type Rec8 or the non-cleavable phospho-mutant Rec8-24A from an estradiol-inducible promoter at anaphase I. Top, immunoblot analysis of whole cell extracts. Separase activity at meiosis II generates cleavage products from wild-type Rec8 but not from Rec8-24A. Bottom, analysis of progression through meiosis by immunofluorescence microscopy of fixed cells ($n = 100$ per time point). Non-cleavable Rec8-24A has no effect on nuclear division at meiosis II. Data are representative of two independent experiments.

Source data are available online for this figure.

© 2021 The Authors

Figure EV5. Localization of Sgol2 in oocytes.

A Oocytes expressing histone H2B-miRFP670 (blue), mScarlet-CenpC (kinetochores, red), and mNeonGreen-Sgol2 (green) were imaged at 5-min intervals from GVBD to metaphase II (12.5 h). Selected frames are shown. Scale bar: 10 μm.

B Chromosomes spreads from oocytes at metaphase II, early anaphase II (chromatids in the process of segregating), late anaphase II (chromatids fully segregated), and metaphase II plus nocodazole were stained for DNA, Sgol2, and kinetochores (ACA) and scored for the presence/absence of Sgol2 signals. Nocodazole reduces the inter-kinetochore distance of dyad chromosomes, implying that the spreading procedure preserves the configuration of bioriented sister kinetochores. Scale bars: 10 μm. Magnification of insets: 3×.

 © 2021 The Authors

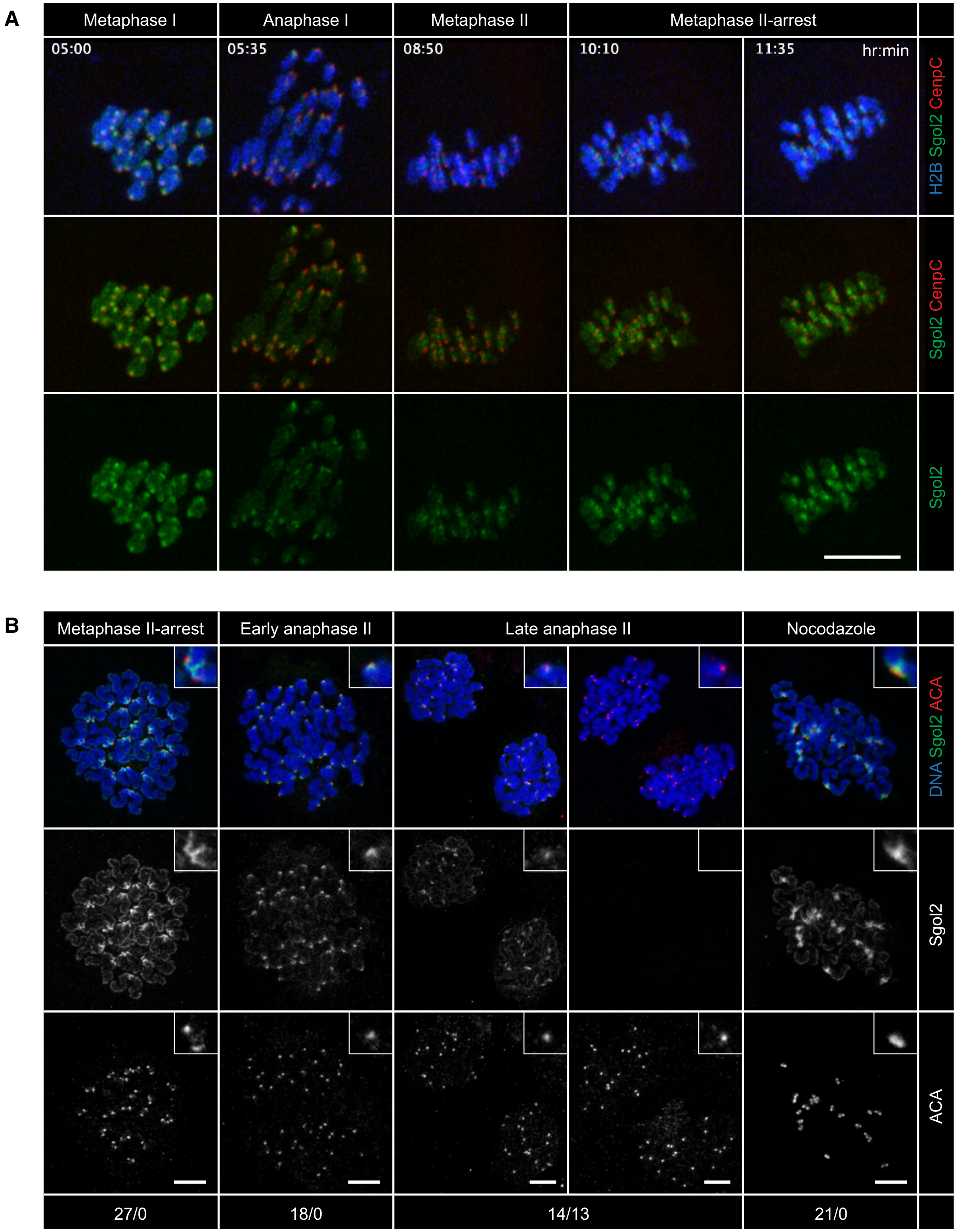
A
Metaphase I
Anaphase I
Metaphase II
Metaphase II-arrest
05:00
05:35
08:50
10:10
11:35
hr:min
H2B Sgol2 CenpC
Sgol2 CenpC
Sgol2
B
Metaphase II-arrest
Early anaphase II
Late anaphase II
Nocodazole
DNA Sgol2 ACA
Sgol2
ACA
27/0
18/0
14/13
21/0

Figure EV5.

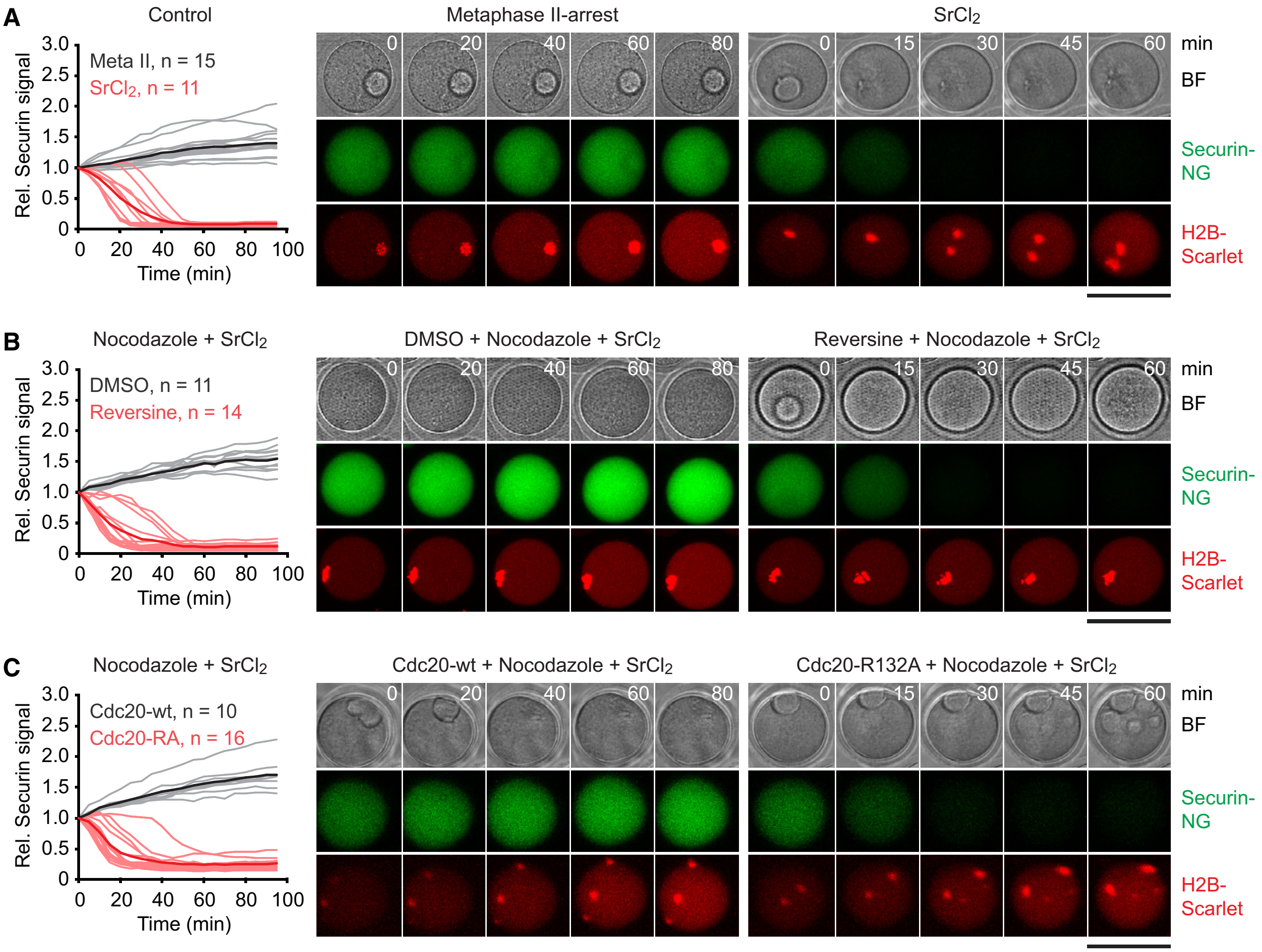

Figure EV6. Imaging of securin to analyze APC/C activity in meiosis II-oocytes.

A–C *In vitro*-matured oocytes were injected with securin-mNeonGreen and histone H2B-mScarlet mRNAs, allowed to progress to the metaphase II arrest, and treated with DMSO (control) or nocodazole for 0.5 h. When indicated, oocytes were treated with reversine or injected with Cdc20-R132A mRNA to inactivate the SAC. Finally, oocytes were exposed to $SrCl_2$ to inactivate CSF and imaged every 5 min for 95 min. Graphs show securin-mNeonGreen fluorescence intensities of individual oocytes (thin lines) and average intensities (thick lines) normalized to the starting value. Images show representative time-lapse series. BF, bright field. Scale bars: 100 µm. (A) Control oocytes treated with water or $SrCl_2$. (B) Oocytes treated with nocodazole or nocodazole plus reversine were exposed to $SrCl_2$. (C) Oocytes were allowed to express either Cdc20-wt or Cdc20-R132A from injected mRNA for 3 h and then exposed to nocodazole and $SrCl_2$.

Data information: (A–C) are representative of two independent experiments.
Source data are available online for this figure.

 © 2021 The Authors
