# Supplementary material for: Deprotection of centromeric cohesin at meiosis II requires APC/C activity but not kinetochore tension
Source: EMBO J. 2021 Mar 1;40(7):e106812. doi: 10.15252/embj.2020106812 (PMC8013787; doi:10.15252/embj.2020106812)
Supplement: Supplementary file 9 — Source Data for Figure 5 [file EMBJ-40-e106812-s013.zip › EMBOJ-2020-106812_SourceDataForFigure5A_1.pdf]

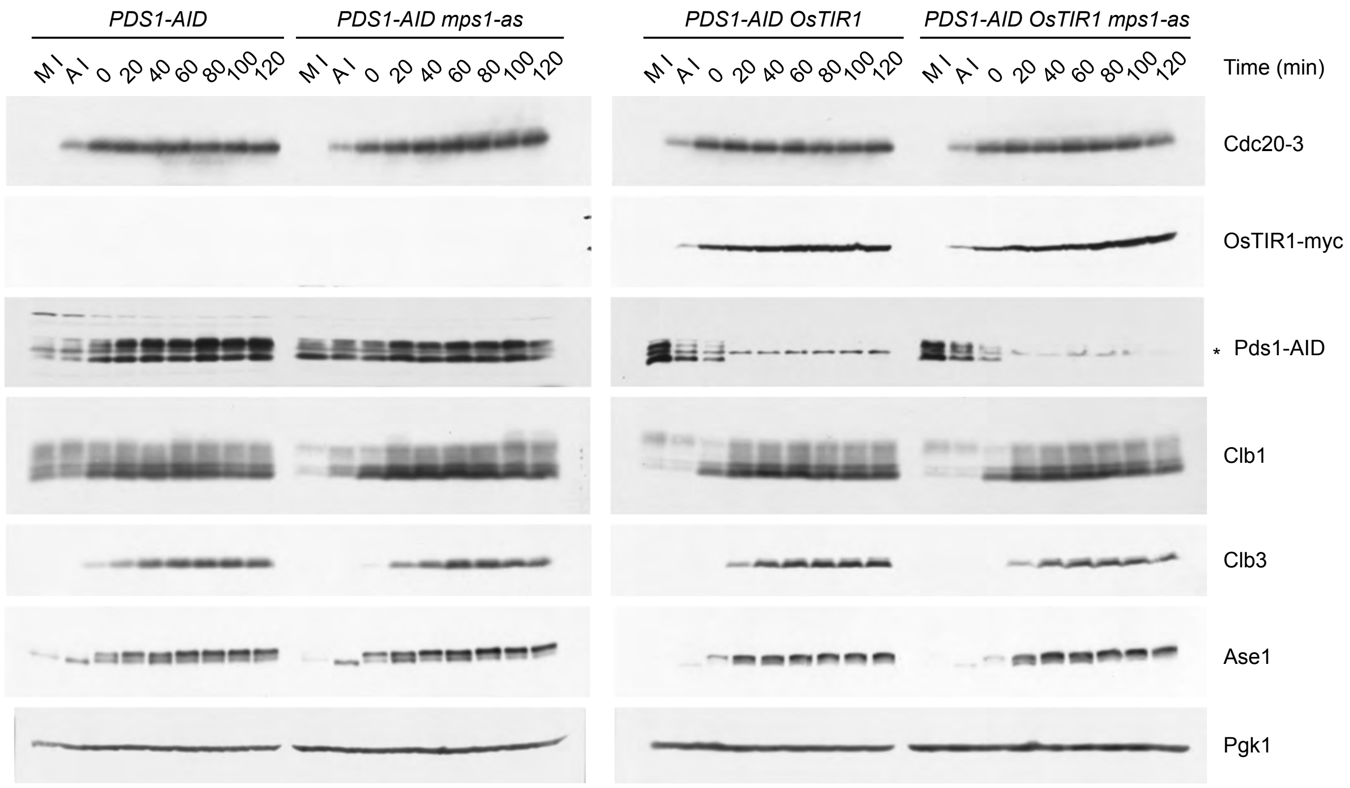

Source data for Figure 5A.

Whole cell extracts were separated in SDS-8% PAA gels and transferred to PVDF membranes. Membranes were horizontally cut into 2-3 slices and incubated with primary antibodies to the indicated proteins. HRP-conjugated secondary antibodies were detected by incubation with ECL reagent and exposure to X-ray film. Membranes with the same protein were processed and exposed together. M I, metaphase I; A I, anaphase I. * non-specific band.
